# Leptin Antagonizes Peroxisome Proliferator-Activated Receptor-**γ** Signaling in Growth Plate Chondrocytes

**DOI:** 10.1155/2012/756198

**Published:** 2012-09-13

**Authors:** Lai Wang, Yvonne Y. Shao, R. Tracy Ballock

**Affiliations:** Orthopaedic and Rheumatologic Research Center, Departments of Biomedical Engineering and Orthopaedic, Surgery, A-41, The Lerner Research Institute, The Cleveland Clinic Foundation, 9500 Euclid Avenue, Cleveland, OH 44195, USA

## Abstract

Leptin is an obesity-associated cytokine-like hormone encoded by the *ob* gene. Recent studies reveal that leptin promotes proliferation and differentiation of chondrocytes, suggesting a peripheral role of leptin in regulating growth plate function. Peroxisome proliferator-activated receptor-**γ** (PPAR**γ**) is a transcriptional regulator of adipogenesis. Locally, PPAR**γ** negatively regulates chondrogenic differentiation and terminal differentiation in the growth plate. The aim of this study was to test the hypothesis that leptin may suppress the inhibitory effects of PPAR**γ** on growth plate chondrocytes. Chondrocytes were collected from distal femoral growth plates of newborn rats and were cultured in monolayer or cell pellets in the presence or absence of leptin and the PPAR**γ** agonist ciglitazone. The results show that leptin attenuates the suppressive effects of PPAR**γ** on chondrogenic differentiation and T3-mediated chondrocyte hypertrophy. Leptin treatment also leads to a mild downregulation of PPAR mRNA expression and a significant MAPK/ERK-dependent PPAR**γ** phosphorylation at serine 112/82. Blocking MAPK/ERK function with PD98059 confirmed that leptin antagonizes PPAR**γ** function in growth plate chondrocytes through the MAPK/ERK signaling pathway. Furthermore, leptin signaling in growth plate cells is also negatively modulated by activation of PPAR**γ**, implying that these two signaling pathways are mutually regulated in growth plate chondrocytes.

## 1. Introduction

The process of longitudinal bone growth is under endocrine regulation. Some of the endocrine signals act locally in regulating growth plate chondrocyte proliferation and differentiation [1]. Leptin is a cytokine-like hormone that is the product of the *ob* gene and is expressed predominantly in adipocytes [2]. Leptin controls body fat tissue and body weight by reducing food intake and increasing thermogenesis [3] and functions via the leptin receptor (OB-R), the long form of which (OB-Rb) is the most abundantly expressed and only biologically active isoform [4]. Binding of leptin to its receptor triggers activation of janus kinases (JAKs) [5], leading to phosphorylation and activation of signal transducer and activator of transcription 3 (STAT3) [6]. Suppressor of cytokine signaling 3 (SOCS3) protein acts as a feedback inhibitor of the JAK/STAT3 pathway, inhibiting STAT3 phosphorylation [7].

Growth plate chondrocytes synthesize and secrete leptin and express the leptin receptor OB-Rb [8]. Leptin is involved in bone remodeling and has a direct peripheral effect on growth plate chondrocytes [8]. Organ cultures of mouse mandibular condyles reveal that leptin induces the proliferation and maturation of growth plate chondrocytes and stimulates endochondral bone growth directly at the level of the bone growth centers [9]. Studies of leptin-deficient *ob/ob* mice demonstrate that lack of leptin protein not only causes obesity in mice [2], but also results in disturbed columnar structure, decreased type X collagen expression, increased apoptosis, and premature mineralization in the growth plates [10]. Administration of leptin to *ob/ob* mice increases bone growth as well as indices of bone formation [2, 10]. Previously, we also reported that leptin synergizes with thyroid hormone in modulating terminal differentiation of growth plate chondrocytes [11], suggesting that peripheral leptin signaling plays an essential role in endochondral ossification at the growth plate. 

PPAR*γ* is a key transcriptional regulator of adipocyte differentiation. It regulates metabolism and storage of fat and is thought to be involved in the development of high fat diet-induced obesity [12]. Our previous studies revealed that PPAR*γ* is expressed in growth plate chondrocytes, and activation of PPAR*γ* promotes adipogenic transdifferentiation of growth plate chondrocytes, while attenuating both chondrogenic differentiation and terminal differentiation [13, 14]. 

Since leptin and PPAR*γ* are both localized in growth plate cartilage and locally modulate chondrocyte function, the object of this study was to investigate the interaction between these two signaling pathways in growth plate chondrocytes. We hypothesized that leptin might prevent the inhibitory effects of PPAR*γ* on chondrogenic differentiation and terminal differentiation of growth plate cells. 

## 2. Materials and Methods

### 2.1. Cell Culture

Chondrocytes were isolated from the distal femoral growth plates of 3-day old neonatal Sprague-Dawley rats by sequential digestion in trypsin/EDTA (Invitrogen, Carlsbad, CA) for 1 h at 37°C, followed by 0.3% collagenase type I (Worthington, Lakewood, NJ) for 4 h at 37°C [15]. Cells were resuspended in DMEM/F12 medium (Invitrogen) supplemented with a defined media supplement (ITS+1, Sigma, St. Louis, MO) and plated in monolayer at a density of 5 × 10^5^ cells/cm^2^, or in a pellet culture of 1 × 10^5^ cells/mL. Tri-iodothyronine (T3, Sigma), leptin (Sigma), and ciglitazone (BioMol, Plymouth Meeting, PA) were added to the medium at concentrations of 100 ng/mL, 1 *μ*g/mL, and 10 *μ*M, respectively, except where specifically indicated. The MAPK/ERK inhibitor PD98059 (20 *μ*M, Cell Signaling Technology, Danvers, MA) and the JNK inhibitor SP600125 (10 *μ*M, Cell Signaling) were added to the medium 30 min before the leptin treatment.

Recombinant adenovirus carrying PPAR*γ*1 (Ad-PPAR*γ*) was kindly provided by Dr. Jameson (Northwestern University Medical School, Chicago, IL) [16] and was used at an MOI (multiplicity of infection) of 100. A structurally similar adenovirus containing the CMV promoter was used as a negative control. 

### 2.2. Transient Transfection

PPAR transcriptional activity was evaluated by cotransfecting the cells with the peroxisome proliferator activator response element (PPRE) reporter plasmid (phRG-TK-PPRE_2_) [14] and PPAR*γ* expression plasmid (pCMX-PPAR*γ*) (provided by R. Evans, Salk Institute, La Jolla, CA) using lipofection (Fugene 6, Roche, Indianapolis, IN) [14]. The transfection mixture was replaced the following day with original medium containing leptin and/or ciglitazone. After 48 hours, cells were harvested and assayed for luciferase activity using the Dual-Luciferase Reporter Assay System (Promega, Madison, WI). The firefly luciferase expression vector pCMV-Luc (Promega) was used as an internal control. 

### 2.3. Quantitative Real-Time RT-PCR

Total RNA was isolated from cultured growth plate chondrocytes using the RNeasy Kit (Qiagen, Valencia, CA). Reverse transcription was performed using random primers and Superscript III (Invitrogen). Real-time PCR reactions were conducted in an ABI Prism 7700 Sequence Detection System using SYBR Green PCR core reagents (Applied Biosystems, Foster City, CA). The forward and reverse primers for the amplifications are listed below:

 18s: 5′-AGTCCCTGCCCTTTGTACACA-3′ and 5′-GATCCGAGGGCCTCACTAAAC-3′;
 Col2a1: 5′-GGTGGAGCAGCAAGAGCAA-3′ and 5′-CGTCGCCGTAGCTGAAGTG-3′;
 Aggrecan: 5′-CTAGCTGCTTAGCAGGGATAACG-3′ and 5′-CCGCAGAGTCACAAAGACCAA-3′;
 Col10a1: 5′-GATCATGGAGCTCACGGAAAA-3′ and 5′-CCGTTCGATTCCGCATTG-3′;
 PPAR*γ*: 5′-TGACCAGGGAGTTCCTCAAAA-3′ and 5′-AGCAAACTCAAACTTAGGCTCCAT-3′;
 Leptin: 5′-CACACACGCAGTCGGTATCC-3′ and 5′-TGAAGCCCGGGAATGAAGT-3′;
 Leptin receptor (Ob-Rb): 5′-CTTAAGAACCCCTTCAAGAATTATGACT-3′ and 5′-GGGCAGAGGCAAATCATCTATAAC-3′;
 SOCS3: 5′-CCTCAAGACCTTCAGCTCCAA-3′ and 5′-TCCGCTCTCCTGCAGCTT-3′.

### 2.4. Alkaline Phosphatase (ALP) Activity Assay

Chondrocyte pellets were homogenized and alkaline phosphatase activity determined as previously described using *p*-nitrophenyl phosphate (Sigma) as a substrate [15]. One unit of alkaline phosphatase was defined as the enzyme activity that liberated 1 *μ*mol *p*-nitrophenol per 30 min at 37°C per mg of protein.

### 2.5. Histochemical Stainings

Histological stainings were performed on the chondrocytes cultured in monolayer. Cells were fixed in 3.7% formaldehyde at room temperature for 10 min and rinsed with PBS. For Alcian blue staining, cells were stained with a 4 : 1 ratio of 0.1 M HCl/0.5% Alcian blue stock [0.5% Alcian blue 8GX (Sigma) in 95% ethanol] overnight at 37°C in a humidified atmosphere. For alkaline phosphatase staining, cells were stained in the dark for 30 min in a 0.1 M Tris-HCl solution (pH 8.5) containing 0.2 mg/mL of Napthol AS-MX phosphate (Sigma) and 0.6 mg/mL of Fast Blue BB salt (Sigma). 

### 2.6. Immunoblotting

Whole cell extracts were prepared from growth plate chondrocytes using RIPA buffer. An equal amount of protein was subjected to SDS-PAGE and transferred onto nitrocellulose membranes. The blots were incubated with anti-phospho-PPAR*γ* (Ser112 of PPAR*γ*2 and Ser82 of PPAR*γ*1) (Assay Biotechnology Inc, Sunnyvale, CA), anti-PPAR*γ* (H100, Santa Cruz Biotechnology, Santa Cruz, CA), anti-phospho-p44/42 MAPK (ERK1/2) (Thr202/Tyr204) (Cell Signaling), anti-p44/42 MAPK (ERK1/2) (Cell Signaling), anti-phospho-Stat3 (Tyr705) (Cell Signaling), anti-Stat3 (Cell Signaling), and anti-*β*-actin (Sigma), followed by a HRP-conjugated secondary antibody. Immunoreactive proteins were visualized by Western Blotting Chemiluminescence Luminol Reagent (Santa Cruz). 

### 2.7. Statistical Analysis

The results are represented as mean ± standard deviation. Data were analyzed by one-way ANOVA with post-hoc Tukey's HSD test or paired Student's *t*-test using JMP 8 software (SAS Institute Inc., Cary, NC). Statistical significance was set at *P* < 0.05.

## 3. Results

### 3.1. Leptin Attenuates PPAR*γ*-Induced Inhibition in Chondrogenic Differentiation and T3-Mediated Hypertrophy

**Figure 1:**
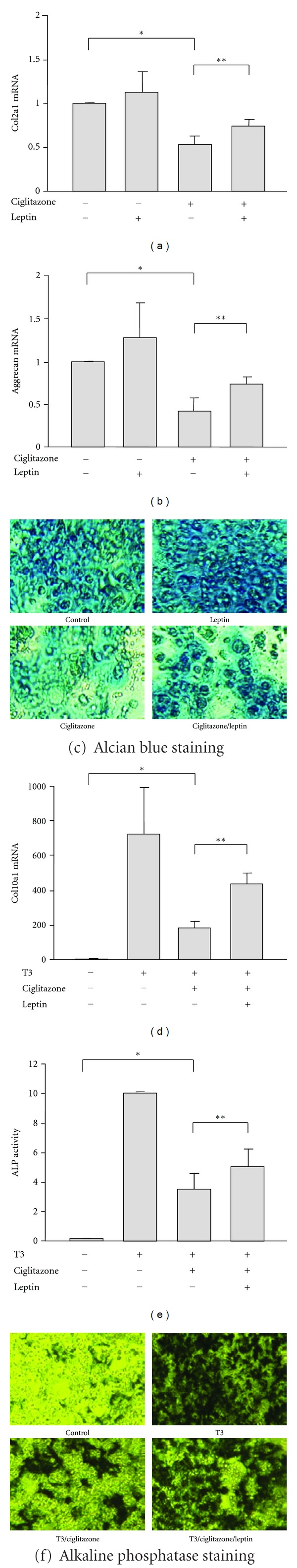
Leptin suppresses the effects of PPAR*γ* on chondrogenic differentiation and chondrocyte hypertrophy in growth plate chondrocytes. ((a), (b)) Quantitative real-time RT-PCR analysis of Col2a1 (a) and aggrecan (b) mRNA expression in chondrocytes treated with ciglitazone and/or leptin for 5 days. *P < 0.05 versus the expression in control cells. **P < 0.05 versus the expression in the cells treated with ciglitazone alone. (c) Alcian blue staining of growth plate chondrocytes in monolayer cultures after 5 days of treatment with ciglitazone and/or leptin. ((d), (e)) Expression of Col10a1 mRNA expression (d) and alkaline phosphatase activity (e) of growth plate chondrocytes treated with ciglitazone and/or leptin for 5 days. *P < 0.05 versus the cells treated with T3 alone. **P < 0.05 versus the chondrocytes treated with both T3 and ciglitazone. (f) Alkaline phosphatase staining of chondrocytes cultured in monolayer and treated with ciglitazone and/or leptin for 5 days.

Incubating the growth plate chondrocytes with the PPAR*γ* agonist ciglitazone for 5 days decreased both Col2a1 and aggrecan mRNA expression (Figures 1(a) and 1(b)), and reduced Alcian blue staining, an index for proteoglycan matrix accumulation (Figure 1(c)). Coaddition of leptin reduced the ciglitazone-induced inhibition of these chondrogenic differentiation markers. As previously observed [14], ciglitazone also inhibited T3-mediated chondrocyte hypertrophy, as shown by decreased Col10a1 mRNA expression (Figure 1(d)) and ALP activity (Figure 1(e)), as well as reduced ALP staining (Figure 1(f)). These decreases were also alleviated by coincubation with leptin (Figures 1(d)–1(f)).

### 3.2. Leptin Inhibits PPAR*γ* Signaling by Activating MAPK/ERK Pathway

**Figure 2:**
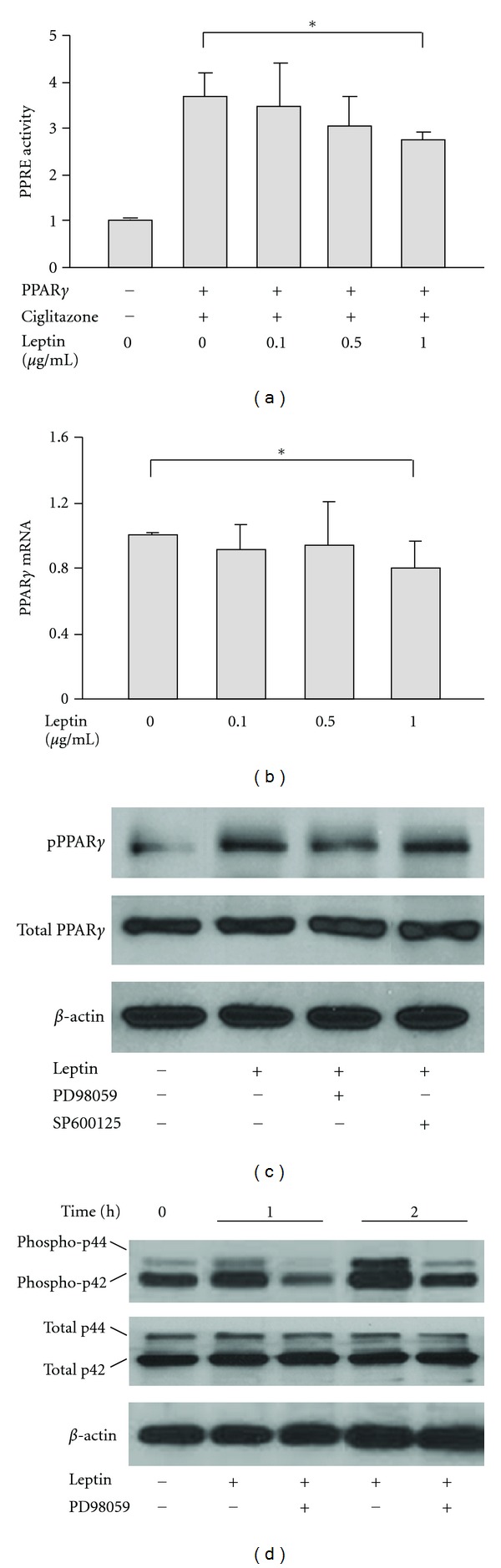
Leptin inhibits PPAR*γ* signaling in growth plate chondrocytes by enhancing PPAR*γ* phosphorylation via MAPK/ERK pathway. (a) PPAR*γ*-mediated PPRE transcriptional activity in chondrocytes treated with leptin at a concentration ranged from 0.1 to 1 *μ*g/mL. *P < 0.05 versus the samples untreated with leptin. (b) PPAR*γ* mRNA expression of growth plate chondrocytes in response to leptin treatment. (c) Immunoblotting analysis of the levels of total and phosphorylated (Ser112/82) PPAR*γ* protein in chondrocytes treated with leptin for 2 h. For ERK or JNK inhibition experiments, the cells were preincubated with PD98059 or SP600125 for 30 min before leptin treatment. (d) Expression of total and phosphorylated ERK proteins (ERK1/2) in growth plate chondrocytes treated with leptin for 1 h and 2 h.

In the dose dependent experiments, leptin was used at a range from 0.1 *μ*g/mL to 1 *μ*g/mL. Treatment with 1 *μ*g/mL of leptin modestly inhibited PPAR*γ*/ciglitazone-increased PPRE transcriptional activity (Figure 2(a)) and downregulated PPAR*γ* mRNA expression (Figure 2(b)). 

Immunoblotting analysis showed that leptin treatment led to no significant change in total PPAR*γ* protein level, but did lead to an increase in phosphorylated PPAR*γ*, which was detected by an antibody directly against the phosphorylation site at Ser112/82 of PPAR*γ* (Figure 2(c)). These leptin-induced increases in phosphorylated PPAR*γ* were blocked by coincubation with the MAPK/ERK inhibitor PD98059, but not by the JNK inhibitor SP600125 (Figure 2(c)). 

Activation of MAPK/ERK signaling by leptin was confirmed by examining the phosphorylation of ERK proteins p42 and p44 (ERK1/2) using immunoblotting. Cell lysates were collected from chondrocytes treated with leptin for 1 h and 2 h. Compared with the leptin untreated controls, leptin increased the levels of both phosphorylated p42 and p44 ERK, and these increases were blocked by PD98059 (Figure 2(d)).

### 3.3. Leptin Regulates PPAR*γ* Effects on Growth Plate Chondrocytes through the MAPK/ERK Signaling Pathway

**Figure 3:**
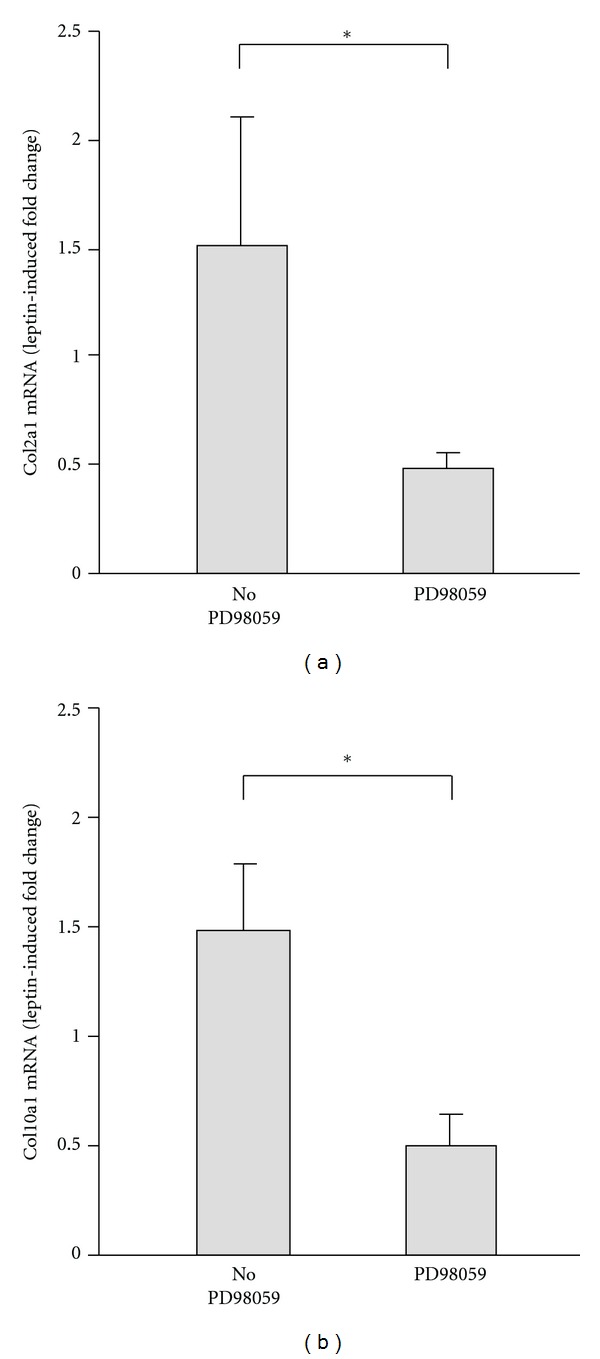
Leptin suppresses the PPAR*γ* inhibitory effects on growth plate chondrocytes by activating MAPK/ERK signaling pathway. ((a), (b)) Gene expression of chondrogenic differentiation marker Col2a1 (a) and terminal differentiation marker Col10a1 (b) in chondrocytes treated with ciglitazone and leptin for 5 days in the presence and absence of PD98059. T3 was added to induce the chondrocyte terminal differentiation. Data are presented as the leptin-induced fold changes in ciglitazone-treated chondrocytes, normalized to the expression in cells treated with ciglitazone alone. *P < 0.05 versus expression changes in the chondrocytes untreated with PD98059.

To evaluate the role of MAPK/ERK signaling in the leptin-induced suppression of PPAR*γ* inhibitory effects on growth plate cell chondrogenic differentiation and chondrocyte hypertrophy, MAPK/ERK inhibitor PD98059 was presupplemented in the medium 30 min before the treatment with leptin and/or ciglitazone for 5 days. As shown in Figures 3(a) and 3(b), PD98059 abolished the leptin-induced increases of Col2a1 and Col10a1 mRNA expression in ciglitazone-treated growth plate chondrocytes (Figures 3(a) and 3(b)).

### 3.4. Leptin Signaling Is Regulated by PPAR*γ* in Growth Plate Chondrocytes

**Figure 4:**
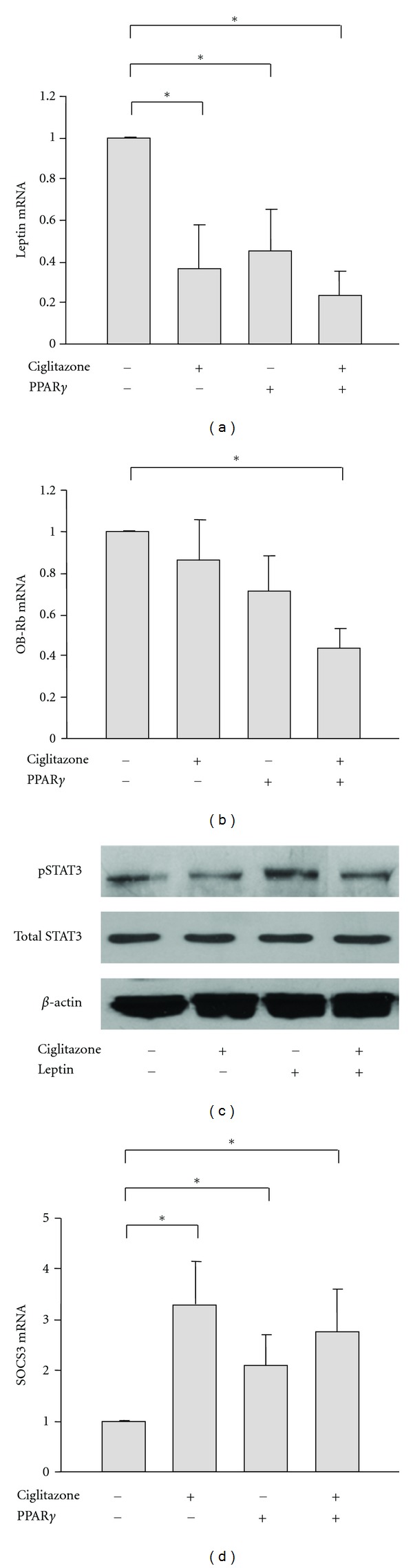
. Leptin signaling is regulated by PPAR*γ* activation in growth plate chondrocytes. (a) and (b) Real-time RT-PCR analysis of gene expression of leptin ligand (a) and leptin receptor Ob-Rb (b) in growth plate chondrocytes treated with ciglitazone and/or Ad-PPAR*γ* for 5 days. *P < 0.05 versus the expression in control cells. (c) Immunoblotting analysis of the levels of total and phosphorylated STAT3 in the lysates of chondrocytes treated with ciglitazone and/or leptin for 5 days. (d) SOCS3 mRNA expression in growth plate chondrocytes treated with ciglitazone and/or Ad-PPAR*γ* for 5 days. *P < 0.05 versus the expression in control cells.

The influence of PPAR*γ* on leptin signaling was analyzed in growth plate chondrocyte pellet cultures treated with ciglitazone and/or infected with Ad-PPAR*γ*. Treatment of growth plate cells with ciglitazone and/or Ad-PPAR*γ* for 5 days led to decreases in leptin mRNA expression (Figure 4(a)). Leptin receptor (Ob-Rb) expression was also downregulated when treating the cells with both ciglitazone and Ad-PPAR*γ* (Figure 4(b)). Immunoblotting analysis of the chondrocytes treated with leptin and/or ciglitazone for 5 days demonstrated that leptin increased phosphorylation of STAT3 (Figure 4(c)). Incubation of the cells with ciglitazone decreased the phosphorylation of STAT3 and inhibited leptin-induced STAT3 activation (Figure 4(c)). Expression of SOCS3, a leptin signaling inhibitor, was increased after ciglitazone and/or Ad-PPAR*γ* treatment (Figure 4(d)).

## 4. Discussion

Childhood obesity has become one of the most serious public health problems in recent decades [17]. Several pediatric orthopedic conditions are known to be related to obesity and involve the growth plates, including slipped capital femoral epiphysis (SCFE), adolescent Blount's disease, and increased risk of growth plate fracture [18, 19]. Disorganization of the normal columnar architecture and impaired differentiation into hypertrophic cells have been observed in the growth plates of both SCFE and Blount's disease patients [20], suggesting that dysfunction of the growth plate in obese children may contribute to the skeletal developmental abnormalities in addition to the mechanical stress resulting from increased body weight. 

The relationship between leptin and pediatric obesity has been widely reported. Similar to mouse models, mutations in human leptin and/or leptin receptor genes are associated with early-onset childhood obesity [21]. Leptin levels are increased in obese children in direct proportion to the increase in body mass index [22]. The elevated circulating levels of leptin are thought to be important for the obese children to have normal rates of longitudinal growth, despite of their low levels of growth hormone [23]. 

In this study, we investigated the interaction between leptin and PPAR*γ*, another important regulator of adiposity and energy balance. We show that addition of leptin partially releases the suppressive effect of PPAR*γ* on chondrogenic differentiation and terminal differentiation in growth plate chondrocytes. The finding that addition of leptin only modestly decreases PPAR*γ* expression at the mRNA level suggests that leptin might suppress PPAR*γ* activity also by posttranslational modifications. 

Genomic activity of PPAR*γ* is regulated by various cellular processes. PPAR*γ* is a phosphoprotein which is phosphorylated by MAPK signaling [24, 25]. Epidermal growth factor and platelet-derived growth factor have been reported to decrease the transcriptional activity of PPAR*γ* by increasing its phosphorylation through MAPK signaling [25]. Both MAPK/ERK and MAPK/JNK signaling can phosphorylate PPAR*γ* at a consensus MAPK phosphorylation site, serine 82 of mouse PPAR*γ*1 and serine 112 of mouse PPAR*γ*2 [26]. MAPK-mediated phosphorylation inhibits PPAR*γ* transactivation function by attenuating PPAR*γ* ligand-binding affinity [27], PPAR*γ* nuclear export [28], and PPAR*γ* inactivation by proteasomal degradation [29].

Our present study in rat growth plate chondrocytes reveals that leptin induces PPAR*γ* phosphorylation at serine 112/82. The fact that this PPAR*γ* phosphorylation is blocked by the ERK inhibitor PD98059 but not the JNK inhibitor SP600125 indicates that MAPK/ERK but not MAPK/JNK signaling is involved in the regulation of PPAR*γ* by leptin in growth plate chondrocytes. These data are in agreement with findings in the ATDC5 chondrogenic cell-line, in which leptin has been reported to increase phosphorylation of ERK1/2 in a time- and dose-dependent manner, but not phosphorylation of JNK [30]. Furthermore, inhibition of MAPK/ERK by PD98059 abolishes the preventive effects of leptin on PPAR*γ*-reduced chondrogenic differentiation and terminal differentiation, implying that the effects of leptin on PPAR*γ* function in growth plate chondrocytes may result from MAPK/ERK-mediated PPAR*γ* phosphorylation. 

The MAPK/ERK pathway plays a role in chondrocyte differentiation and proliferation by mediating the upregulation of Sox9 and cyclin D1 expression [31, 32] and also has been reported to be a negative regulator of endochondral bone growth by inhibiting hypertrophic differentiation of chondrocytes [33, 34]. We previously reported that leptin promotes growth plate chondrocyte proliferation and terminal differentiation in part through IGF-1/IGF1R and Wnt/*β*-catenin signaling pathways [11]. The results of this study suggest that activation of MAPK/ERK by leptin may also promote chondrocyte proliferation and contribute to chondrocyte terminal differentiation by enhancing the number of proliferative cells. The finding that leptin-induced changes in Col2a1 and Col10a1 expression in the ciglitazone-treated chondrocytes are decreased by PD98059 indicate that leptin-activated MAPK/ERK signaling is involved in the inhibition of PPAR*γ* activity and the negative effects of PPAR*γ* on growth plate chondrocytes. 

Activation of PPAR*γ* has been reported to inhibit leptin gene expression in adipocytes [35, 36]. Heterozygous PPAR*γ*-deficient mice exhibit high bone mass with higher leptin levels than wildtype littermates [37]. The inhibition of leptin by PPAR*γ* may result from functional antagonism of liganded PPAR*γ* on the CCAAT/enhancer binding protein *α* (C/EBP*α*) transactivation of the leptin promoter [36]. 

In our study, activation of PPAR*γ* also negatively regulates leptin signaling. PPAR*γ* and its agonist ciglitazone downregulate leptin, and its receptor mRNA expression, inhibit leptin-induced STAT3 phosphorylation and activation and increase STAT3 inhibitor SOCS3 expression. These findings indicate that PPAR*γ* and leptin signaling pathways are mutually regulated in growth plate chondrocytes. The imbalance between the levels of PPAR*γ* and leptin may facilitate the dysfunction of the growth plate observed in obese children.

## Abbreviations

PPAR*γ*: Peroxisome proliferator-activated receptor-*γ*
MAPK: Mitogen-activated protein kinase
ERK: Extracellular signal-regulated kinase
JNK: c-Jun N-terminal kinase
JAK/STAT3: Janus kinase/signal transducer and activator of transcription 3
SOCS3: Suppressor of cytokine signaling 3.
